# Interrelationships Between Patients’ Data Tracking Practices, Data Sharing Practices, and Health Literacy: Onsite Survey Study

**DOI:** 10.2196/18937

**Published:** 2020-12-22

**Authors:** Yuhan Luo, Chi Young Oh, Beth St Jean, Eun Kyoung Choe

**Affiliations:** 1 College of Information Studies University of Maryland College Park, MD United States; 2 Chicago State University Chicago, IL United States

**Keywords:** consumer health informatics, patient-generated health data, self-tracking, doctor-patient data sharing, health literacy, surveys and questionnaires

## Abstract

**Background:**

Although the use of patient-generated data (PGD) in the optimization of patient care shows great promise, little is known about whether patients who track their PGD necessarily share the data with their clinicians. Meanwhile, health literacy—an important construct that captures an individual’s ability to manage their health and to engage with their health care providers—has often been neglected in prior studies focused on PGD tracking and sharing. To leverage the full potential of PGD, it is necessary to bridge the gap between patients’ data tracking and data sharing practices by first understanding the interrelationships between these practices and the factors contributing to these practices.

**Objective:**

This study aims to systematically examine the interrelationships between PGD tracking practices, data sharing practices, and health literacy among individual patients.

**Methods:**

We surveyed 109 patients at the time they met with a clinician at a university health center, unlike prior research that often examined patients’ retrospective experience after some time had passed since their clinic visit. The survey consisted of 39 questions asking patients about their PGD tracking and sharing practices based on their current clinical encounter. The survey also contained questions related to the participants’ health literacy. All the participants completed the survey on a tablet device. The onsite survey study enabled us to collect ecologically valid data based on patients’ immediate experiences situated within their clinic visit.

**Results:**

We found no evidence that tracking PGD was related to self-reports of *having sufficient information to manage one’s health*; however, the number of data types participants tracked positively related to their self-assessed *ability to actively engage with health care providers*. Participants’ data tracking practices and their health literacy did not relate to their data sharing practices; however, their ability to engage with health care providers positively related to their willingness to share their data with clinicians in the future. Participants reported several benefits of, and barriers to, sharing their PGD with clinicians.

**Conclusions:**

Although tracking PGD could help patients better engage with health care providers, it may not provide patients with sufficient information to manage their health. The gaps between tracking and sharing PGD with health care providers call for efforts to inform patients of how their data relate to their health and to facilitate efficient clinician-patient communication. To realize the full potential of PGD and to promote individuals’ health literacy, empowering patients to effectively track and share their PGD is important—both technologies and health care providers can play important roles.

## Introduction

### Background

The prevalence of mobile health apps and wearable devices has enabled patients to track a variety of patient-generated data (PGD) outside the clinic, ranging from biometrics (eg, blood pressure, heart rate) to their everyday activities (eg, exercise, sleep) [1]. Researchers have found that PGD are a valuable source of information for clinicians because they can use the data for diagnosis and treatment [2-6]. Data-driven consultation has the potential to optimize patient care by leveraging effective PGD sharing [7,8]. However, little is known about whether and to what extent patients who track their PGD actually share their data with clinicians in the clinic, which is critical to fully leveraging the potential of PGD to improve health outcomes.

In prior studies that have examined how patients leverage PGD, a key related concept that has often been neglected is health literacy—the cognitive and social skills to gain access to, understand, and use health information in ways that maintain good health [9]. As a multidimensional concept, health literacy characterizes not only an individual’s ability to read and understand health information but also the ability to manage one’s health and engage with health care providers [9-11]. Although health literacy has been studied in public health for decades, this concept has rarely been examined within the context of studies focusing on PGD tracking and sharing.

Previous studies have found that through self-tracking of their PGD, patients can develop the skills necessary to interpret health information [12] and to communicate with clinicians [13]. In this light, we aim to understand whether patients’ self-tracking practices, as a way to help them gain knowledge about their health [14-18], relate to their health literacy and whether patients’ health literacy, as an important skill to fully engage with health care providers [10,11,19], relates to patients’ practice of sharing their PGD with clinicians.

### Related Work

#### PGD Tracking

According to previous research, PGD is defined as “health-related data created, recorded, or gathered by patients (or by family members or other caregivers) to help address their health concerns” [1,15]. These data can be collected through automated tracking devices (eg, mobile phones, wearable devices), manual journaling, questionnaires, clinic tests (eg, laboratory results), and tests ordered by patients (eg, 23andMe [20]). Researchers have found that tracking PGD is an effective means for personal health management [12,21,22]. For example, people with diabetes who monitor their glucose levels go through several learning phases to develop an understanding of how their daily activities affect their health [12]. Similarly, people with irritable bowel syndrome (IBS), who track their food and symptom data, conduct self-experiments to identify the types of food that trigger their IBS symptoms [22]. 

Despite its benefits, PGD tracking can be detrimental under some circumstances. For example, tracking calorie intake can exacerbate negative thoughts and behaviors of patients with eating disorders [23]. In this case, clinicians often recommend to such patients that they manage their eating practices by tracking their feelings and reflections about food and by focusing on the positive sides [3,24]. Therefore, when encouraging patients to track and share their PGD, it is important to tailor the tracking plans of individuals to their tracking needs and their specific health conditions.

#### PGD Sharing

This study was conducted in the clinic where patients were likely to share their PGD with clinicians in person [1]. From the perspective of clinicians, PGD can support in the diagnosis and delivery of personalized treatment [4,6,13]. For example, sleep data are used by clinicians conducting cognitive behavioral therapy for insomnia to inform *sleep prescriptions* [4]. Similarly, food intake data are an important source for diagnosing and treating dietary problems [3]. Although many clinicians acknowledge that having patients share their PGD can improve the quality of care that they can provide [4,6,15,25-27], their views on sharing PGD are not always positive because of their increased liability, lack of time, doubts regarding data accuracy, integration difficulties, and security concerns [6,28-31].

Patients’ preferences for sharing PGD also vary: some patients consistently share their data to keep their clinicians informed [1], some resist sharing their data because of privacy concerns or fear of being judged [3], and others share their PGD, but only to the extent to which they trust their clinicians [4,32]. Although researchers have examined patients’ data tracking and sharing practices, we lack empirical data on whether and to what extent patients actually share their self-tracking data during clinical visits and the factors influencing their data sharing practices.

#### Health Literacy

Health literacy has been declared a national priority in the United States [33]. Previous studies have found that patients with higher health literacy are more likely to be informed about their health [34], to engage in healthy behaviors [35,36], and to feel comfortable while communicating with health care professionals [37,38]. Therefore, health literacy has been used as a critical measure for evaluating the effectiveness of health care technologies [18,39].

To assess patient health literacy, researchers have developed various instruments. The most widely used measures include the Test of Functional Health Literacy in Adults (TOFHLA) [40], the eHealth Literacy Scale (eHEALS) [41], and the Health Literacy Questionnaire (HLQ) [11]. The TOFHLA captures the reading comprehension and numeracy of patients within health care contexts [40], whereas the eHEALS measures the skills of patients related to finding, evaluating, and applying electronic health information to health problems [41]. We chose the HLQ because it captures multiple independent constructs regarding different aspects of health literacy that can yield actionable insights [42,43]. According to the grounded psychometric development and validation of the HLQ [11], each of the 9 metrics in HLQ provides unique insights into 9 areas of health literacy and therefore can be used separately when all the questions in 1 metric are asked (each metric has 4-5 questions). To keep the survey to a reasonable length, we included 2 of the 9 constructs included in the HLQ—*having sufficient information to manage one’s health (HSI)* and *the ability to actively engage with health care providers (AE)*—that are particularly relevant to PGD tracking and sharing [11]. Individuals with higher levels of HSI feel *confident that they have information they need to live with and manage their health*; those with lower levels of HSI feel they lack *knowledge in managing their health*. Individuals with higher levels of AE are *proactive about their health and feel in control in relationships with health care providers*; those with lower levels of AE are *passive in their approach to health care and do not have a sense of agency in interactions with clinicians* [11].

### Research Questions

Although health literacy has not yet been explicitly considered in studies related to PGD, researchers have found that tracking PGD can increase people’s awareness of their health [14] and enable them to better answer their clinicians’ questions [15,44,45]. Therefore, we speculate that tracking PGD may relate to one’s perception regarding HSI and AE. In addition, we assume that patients who shared their PGD in the clinic had tracked their PGD before and were equipped with sufficient HSI and AE. For example, a high level of HSI could enable patients to share their health data with clinicians by equipping patients with sufficient knowledge about their health and a high level of AE could facilitate patients’ data sharing as a way of engaging with clinicians. Thus, we investigate the following research questions (RQs):

- RQ1: Do patients’ self-tracking practices relate to their HSI and AE?
- RQ2: Do patients’ self-tracking practices, along with their HSI and AE, relate to their practices of sharing their PGD with their clinicians?
- RQ3: What benefits and barriers do patients encounter with regard to sharing their PGD with their clinicians?

Although most PGD-related studies have examined patients’ experiences retrospectively after some time had passed since their clinic visit [1,13], we sought to gather the immediate perceptions of patients regarding their experiences of meeting with a clinician. We surveyed 109 patients who had just met with a clinician at a university health center (UHC), asking them about their practices of tracking and sharing PGD and assessing their health literacy (ie, HSI and AE). Conducting the survey onsite at the UHC enabled us to collect the experiences of participants from their current clinic visit, which helped reduce recall bias and enabled us to gather ecologically valid data. 

## Methods

### Survey Design and Measures

Throughout the survey, we used the term *personal health data* instead of PGD to aid participants’ understanding. At the beginning of the survey, we defined *personal health data* as *health-related data that people keep track of about themselves, such as exercise (eg, step count, miles run), sleep, diet, heart rate, and blood pressure*. Table 1 and Textbox 1 describe our survey measures, including how we defined and assessed each construct.

**Table 1:** Quantitative survey measures.

Quantitative measures and descriptions		Scale or categories
**Health status**		
	Self-reported health status of participants	1: very poor to 5: excellent
**Tracking practices**		
	Number of data types: the total number of different types of health data participants reported being tracked	0: track nothing to 9: track 9 types of PGD^a^
	Tracking group: how participants kept track of their PGD	Nontrackers: tracked nothing Memory trackers: tracked PGD relying solely on their memory Tool trackers: tracked PGD using at least one type of tool (eg, paper, mobile app)
**Sharing practices**		
	Data sharing: whether participants had shared their health data with clinicians during their clinic visit	Yes or no
	Willingness to share: how likely participants said they would be willing to share their data with clinicians in the future	1: very unlikely to 5: very likely
**Health literacy**		
	Having sufficient information to manage one’s health	Averaged score from 4 measures on a scale of 1: *strongly disagree* to 4: *strongly agree* (Multimedia Appendix 1: questions 16-19) [11]
	The ability to actively engage with health care providers	Averaged score from 5 measures on a scale of 1: *cannot do* to 5: *very easy* (Multimedia Appendix 1: questions 20-24) [11]

^a^PGD: patient-generated data.

**Qualitative survey measures.**

- **Data tracked:**
  The types of patient-generated data (PGD) that participants reported tracking (multiple-choice question with an option to write in additional data types)
- **Data shared:**
  The types of PGD that participants reported sharing during the particular appointment with the clinician (multiple-choice question with an option to write in additional data types)
- **Data wished to track and be shared:**
  The types of PGD that participants thought would have been helpful if they had tracked and shared them (open-ended question: “List up to 3 types of personal health data that you think it would have been helpful if you had tracked them and shared them with your doctor today”)
- **Sharing benefits:**
  Benefits that patients perceived they would have had by sharing their PGD during the particular encounter (an open-ended question asking participants to explain how sharing particular types of PGD would have been helpful)
- **Sharing barriers:**
  Barriers that patients encountered in sharing their PGD during their visit to the clinic (multiple-choice question: “what barriers (if any) did you have when sharing your PGD with your doctor?” and a follow-up question asking for further explanation)

In addition, we collected participants’ (1) demographic information (eg, age, gender, occupation, educational attainment, household income, first language), (2) access to technology (eg, internet, mobile phone), and (3) details of their meeting with the clinician (eg, reasons for visit, satisfaction). The survey consisted of 39 questions (Multimedia Appendix 1) and was administered using Qualtrics [46] on a tablet device we provided.

### Data Collection

Data collection for this study was part of a larger project that involved onsite surveys and follow-up interviews at a UHC. This paper focuses on the survey results. Following the UHC’s guidance, 3 researchers set up a study space next to several clinics, including primary care, women’ s health, behavioral health, immunization or allergy, and alternative medicine. We approached patients as they exited clinic offices and asked whether they had just met with a clinician. If the patient answered *yes*, we invited them to participate in the survey. If they expressed an interest, we then led them to the study space, secured their informed consent, and instructed them to take the survey using a tablet paired with a keyboard. As we had 2 tablets set up in the study space, we were able to host 2 participants at the same time. During the study, 2 to 3 researchers were sitting behind the tablets, and each participant was sitting in front of each tablet to complete the survey. Participants would inform the researchers when they completed the survey. We did not recruit any patients who had visited the UHC for mental health reasons, as per the UHC’s request.

The survey took participants about 4 to 16 minutes to complete (mean 7.60, SD 2.62). Each participant received a US $5 campus dining gift card after completing the survey. The study was approved by the Institutional Review Board at the researchers’ university.

### Data Analysis

To answer RQ1 (“Do patients’ self-tracking practices relate to their HSI and AE?”), we used multivariate multiple regression to examine whether the number of data types participants tracked and their tracking group (ie, nontracker, memory tracker, or tool tracker) were related to their HSI and AE levels. As patients’ health status has been found to highly correlate with their health literacy [34], we controlled their health status as a covariate. In addition, we dummy coded the tracking group using nontrackers as the reference group.

To answer RQ2 (“Do patients’ self-tracking practices, along with their HSI and AE, relate to their practices of sharing data with doctors?”), we used multiple logistic regression to predict whether participants had shared their PGD during a particular visit based on their HSI, AE, the number of data types they tracked, and their tracking group. Using this same set of predictors in addition to whether participants had shared their data, we used multiple linear regression to predict participants’ willingness to share their data with clinicians in the future. 

To answer RQ3 (“What benefits and barriers do patients encounter with regard to sharing PGD with doctors?”), we first analyzed participants’ responses to the questions regarding their perceived benefits and barriers of sharing PGD with clinicians, and then categorized these responses using a bottom-up (inductive) approach. In this manner, we developed a coding dictionary that reflected different sharing-related benefits and barriers. We later reorganized the initial codes into potential themes, which complemented our findings for RQ2 by explaining why participants had or had not shared their health data with their clinician during the particular clinic visit.

## Results

### Participants

In total, 112 patients participated in our study; however, 3 patients were subsequently excluded from our analysis as they had visited the UHC simply to receive a flu shot, fill out a form, or get a massage. Among the remaining 109 patients, 86 (78.9%) were female and 73 (67.0%) were native English speakers, and their ages ranged from 18 to 37 years (mean 21.0, SD 3.82). Most of the participants were college students (77/109, 70.6%), some (18/109, 16.5%) were full-time employees, and the rest (14/109, 12.8%) were unemployed. Except for 2, all participants (107/109, 98.2%) had access to the internet and a mobile phone. Participants reported visiting the clinic for an inquiry or examination for a specific injury, illness, or condition (65/109, 59.6%), a follow-up visit (22/109, 20.2%), a regular checkup (18/109, 16.5%), or something else (4/109, 3.7%).

Of the total, 87.2% (95/109) of participants reported tracking at least one type of PGD. Of these 95 participants, 38 (35%) were *memory trackers* who tracked their health data relying on their memory only and 57 (52%) were *tool trackers* who tracked their data using at least one tool (eg, paper journal, mobile app, website). The remaining 12.8% (14/109) of participants were *nontrackers*, who did not track any PGD. Table 2 provides descriptive statistics for each of the 3 tracking groups.

**Table 2:** Descriptive statistics for the 3 tracking groups: nontrackers, memory trackers, and tool trackers (the scale of having sufficient information to manage one’s health ranges from 1 (strongly disagree that one has sufficient information to manage their health) to 4 (strongly agree that one has sufficient information to manage their health) and the score of one’s ability to actively engage with health care providers ranges from 1 (cannot actively engage with health care providers) to 5 (very easy to actively engage with health care providers), with higher scores indicating greater health literacy; N=109).

Participants’ information		Total or overall mean values	Nontrackers (n=14; 12.8%)	Memory trackers (n=38; 34.9%)	Tool trackers (n=57; 52.3%)
Age, years; mean (SD)		21.0 (3.82)	20.0 (2.70)	20.5 (3.92)	21.0 (4.70)
**Gender, n (%)**					
	Female	86 (78.9)	11 (78.6)	27 (71.1)	48 (84.2)
	Male	23 (21.1)	3 (21.4)	11 (28.95)	9 (15.8)
**Health status, n (%)**					
	Excellent	13 (11.9)	2 (14.3)	7 (18.4)	4 (7.0)
	Very good	27 (24.8)	2 (14.3)	7 (18.4)	18 (31.6)
	Good	50 (45.9)	7 (50.0)	17 (44.7)	26 (45.6)
	Fair	18 (16.5)	3 (21.4)	6 (15.8)	9 (15.8)
	Poor	1 (0.9)	0 (0.0)	1 (2.6)	0 (0.0)
**Reason for visit, n (%)**					
	Inquiry or examination	65 (59.6)	8 (57.1)	25 (65.8)	32 (56.1)
	Follow-up visit	22 (20.2)	2 (14.3)	7 (18.4)	13 (22.8)
	Regular checkup	18 (16.5)	3 (21.4)	5 (13.2)	10 (17.5)
	Other	4 (3.7)	1 (7.1)	1 (2.6)	2 (3.5)
Number of data types tracked, mean (SD)		3.46 (2.3)	0 (0)	3.53 (2.0)	4.26 (2.1)
**Was data shared during the appointment? n (%)**					
	No	16 (14.7)	3 (21.4)	5 (13.2)	8 (14.0)
	Yes	93 (85.3)	11 (78.6)	33 (86.8)	49 (85.96)
Number of data types shared, mean (SD)		2.50 (2.01)	2.64 (2.47)	2.21 (1.38)	2.67 (1.95)
**Likely to share data with clinician in the future? n (%)**					
	Very likely	61 (56.0)	11 (78.6)	22 (57.9)	28 (49.1)
	Somewhat likely	31 (28.4)	0 (0.0)	13 (34.2)	18 (31.6)
	Neutral	11 (10.1)	2 (14.3)	1 (2.6)	8 (14.0)
	Somewhat unlikely	3 (2.8)	0 (0.0)	1 (2.6)	2 (3.5)
	Very unlikely	3 (2.8)	1 (7.1)	1 (2.6)	1 (1.8)
**Health literacy scores, mean (SD)**					
	HSI^a^ (Cronbach α=.86)	3.08 (0.53)	3.11 (0.43)	2.97 (0.57)	2.97 (0.58)
	AE^b^ (Cronbach α=.88)	4.16 (0.55)	4.20 (0.54)	4.16 (0.53)	4.15 (0.61)

^a^HSI: having sufficient information to manage one’s health.

^b^AE: the ability to actively engage with health care providers.

### RQ1: Relationship Between Self-Tracking Practices and Health Literacy of Participants (HSI and AE Scores)

The regression model that predicted HSI based on the participants’ health status, the number of data types they tracked, and their tracking group was significant. However, there were no significant effects of the number of data types tracked or the participant tracking group (Table 3). Using the same independent variables, the regression model that predicted AE was found to be significant, and there was a significant main effect of the number of data types participants tracked. This result indicates that when controlling for participants’ health status and their tracking group, those who tracked more data types tended to have higher AE.

**Table 3:** The multivariate linear regression models predicting having sufficient information to manage one’s health and one’s ability to actively engage with health care providers (the tracking group was dummy coded using nontrackers as the reference group). The model that predicts HSI achieved a power of >0.90, and the other model that predicts AE achieved a power of >0.80. Both models have been tested to ensure the absence of multicollinearity (Variance Inflation Factor <3.69).

Outcome variable		HSI^a^	AE^b^
Adjusted *R*^2^		0.146	0.106
Effect size		0.171	0.119
***F* ratio**		**5.618^c^**	**4.205^d^**
	*P* value	<.001	.002
**Health rating**		**0.225^c^**	**0.200^c^**
	*P* value	<.001	<.001
**Number of data types**		**−0.004**	**0.056^e^**
	*P* value	.87	.04
**Memory trackers**		**−0.154**	**−0.279**
	*P* value	.38	.16
**Tool trackers**		**0.032**	**–0.323**
	*P* value	.86	.11

^a^HSI: having sufficient information to manage one’s health.

^b^AE: the ability to actively engage with health care providers.

^c^*P*<.001.

^d^*P*<.01.

^e^*P*<.05.

### RQ2: Relationships Between PGD Tracking, Health Literacy (HSI and AE Scores), and PGD Sharing in the Clinic

#### Tracking Does Not Always Lead to Sharing

A total of 85.3% (93/109) of our participants, including 11 nontrackers, reported that they had shared their PGD with their clinicians during the particular visit. The logistic regression predicting whether participants had shared their data during the particular visit was not significant (Table 4). This result indicates that whether participants shared their PGD during the visit was unrelated to the number of data types they tracked, their tracking group, and their HSI and AE levels.

**Table 4:** The multiple logistic regression model predicting whether a participant had shared their patient-generated data during the particular clinic visit. The model achieved a power of >0.75 and has been tested to ensure the absence of multicollinearity (Variance Inflation Factor <3.79).

Outcome variable			Data sharing
Nagelkerke *R*^2^			0.105
***F* ratio**			**1.277**
	*P* value	.24	
**HSI^a^**			**0.089**
	*P* value	.88	
**AE^b^**			**−1.175**
	*P* value	.06	
**Number of data types**			**0.264**
	*P* value	.12	
**Memory trackers**			**−0.263**
	*P* value	.78	
**Tool trackers**			**−0.608**
	*P* value	.55	

^a^HSI: having sufficient information to manage one’s health.

^b^AE: the ability to actively engage with health care providers.

#### Health Literacy and Willingness to Share One’s PGD With Clinicians in the Future

The regression model predicting participants’ willingness to share their PGD with clinicians in the future was significant, and there were significant effects of whether participants had shared their PGD during the particular visit and their AE level (Table 5). This indicates that, when controlling for other variables, participants who shared their data during the visit and those with a higher level of AE were more likely to be willing to share their PGD with clinicians in the future.

**Table 5:** The multiple linear regression model predicting a participant’s willingness to share their patient-generated data in the future. The model achieves a power of >0.95 and has been tested to ensure the absence of multicollinearity (Variance Inflation Factor <3.80).

Outcome variable		Sharing willingness
Adjusted *R*^2^		0.178
Effect size		0.217
***F* ratio**		**4.888^a^**
	*P* value	<.001
**Data sharing**		**0.812^b^**
	*P* value	.001
**HSI^c^**		**0.277**
	*P* value	.14
**AE^d^**		**0.482^b^**
	*P* value	.009
**Number of data types**		**0.046**
	*P* value	.31
**Memory trackers**		**−0.182**
	*P* value	.57
**Tool trackers**		**−0.442**
	*P* value	.19

^a^*P*<.001.

^b^*P*<.01.

^c^HSI: having sufficient information to manage one’s health.

^d^AE: the ability to actively engage with health care providers.

### RQ3: Benefits of, and Barriers to, Sharing One’s PGD With Clinicians

When asked to list up to 3 types of PGD that the participants felt would have been helpful if they had tracked and shared with their clinicians, 72.5% (79/109) of our participants provided valid responses. These responses included body measures (eg, blood pressure, glucose level, heart rate, body temperature; 47/79, 60%), food (36/79, 46%), sleep (27/79, 34%), water (25/79, 32%), exercise (19/79, 24%), stress (7/79, 9%), menstrual cycle (6/79, 8%), symptoms (6/79, 8%), mood (3/79, 4%), and others (5/79, 6%). In the subsequent open-ended question, participants explained why these data would have been helpful if they had tracked and shared them: (1) to better inform clinicians in detail about their health condition; (2) to receive more personalized care; and (3) to better articulate their illness and health concerns (Textboxes 2 and 3).

**Benefits of sharing one’s patient-generated data with clinicians.**

- Better inform clinicians of their health condition: Participants felt that sharing their patient-generated data (PGD) is generally helpful for clinicians to understand their health condition and identify any abnormality
  “More info, better diagnosis” (P29)
  “Sharing my body temperature might have helped doctors identify abnormality.” (P48)
  “Mood, sleep, and stress could have been helpful because [the doctor] would understand my illness severity.” (P103)
- Enable clinicians to deliver more personalized care: Participants stated that clinicians could provide better advice or treatments that are more relevant to them
  “If I had tracked and shared my skincare routine, the doctor can offer more personalized advices for me.” (P15)
  “The doctor could have told me if my current numbers [food, water, sleep] were healthy so [I] could adjust [them] accordingly.” (P23)
- Help patients to better articulate their illness and health concerns: Participants who experienced difficulties communicating with their clinicians believed that sharing concrete health data could help them better articulate their symptoms and concerns with evidence
  “I could have proved that I’ve been having fevers for the past couple of days, in addition to not eating, drinking, and sleeping as much as usual.” (P46)
  “Instead of saying ‘I have trouble sleeping until 4am and barely sleep,’ I could have said ‘in the past month I have gotten an average of X hours of sleep per night.’” (P66)

**Barriers to sharing one’s patient-generated data with clinicians.**

- Uncertainty regarding the relevance and usefulness of their data: Participants were not sure if the data they had tracked was relevant, important, and useful in relation to their current health issues
  “I am not sure if some symptoms are relevant to my headache and I don’t want to waste time.” (P82)
  “Sometimes I don't know what aspects of my health are important to share with my doctor in regards to the health issue I'm having at the moment.” (P102)
- Perceived irrelevance of one’s data: Participants considered that their patient-generated data (PGD) was not relevant to their current health concerns
  “My problem is women health problem; therefore, the data is not very relevant and she did not ask either.” (P22)
  “Some data, such as my exercise and sleep schedule, is not relevant.” (P64)
- Not having enough data: Participants were unable to share their PGD because they had not sufficiently tracked their data
  “My blood pressure and heart rate vary quite a bit. I would love to be able to track this but I don’t have a good device to do so.” (P65)
  “I am somewhat not precise with what I tell them, so that could mess with the data they need to help me.” (P106)
- Limited time to spend with their clinician: Participants expressed concerns regarding the limited time they got to spend with their clinicians
  “The time I spend with the doctor is so limited and it doesn’t seem like the doctor found it necessary to ask any more additional information.” (P46)
  “I feel like doctors never have enough time. They’re always in a rush.” (P65)
- Privacy concerns: Participants were concerned about talking to clinicians they were not familiar with and about the privacy of their health data
  “I feel uncomfortable sharing personal info with people I just met.” (P95)
  “Hacking of computer systems makes me very concerned about my privacy.” (P34)
- Fear of being judged: Participants worried about doctors judging them because of unhealthy behaviors
  “I say less than what I have been doing so that I am not get frowned upon.” (P45)
  “I am afraid of sharing my smoking habits with doctors.” (P52)

When asked about the barriers they had encountered with regard to sharing their PGD with the clinician, 42.2% (46/109) of our participants reported that they did not experience any barriers, whereas the other 57.8% (63/109) of our participants described specific barriers. Their barriers included uncertainty about the relevance and usefulness of their data (18/63, 29%), feeling their data were irrelevant (14/63, 22%), lack of adequate data (12/63, 19%), having limited time to spend with the clinician (11/63, 18%), privacy concerns (7/63, 11%), fear of being judged (4/63, 6%), and others (5/63, 8%). In Textboxes 2 and 3, we list and provide descriptions and sample quotes for each of these barriers.

## Discussion

### Reflecting on the Relationships Between PGD Tracking and Health Literacy

Our results show that neither the number of data types that participants tracked nor how they tracked their data relates to their HSI; however, when more types of PGD participants were tracked, their AE tended to be higher. We suspect that as participants tracked more types of PGD, they had more information about their health in mind. Armed with this information, participants could more actively engage with their clinicians, articulate their health concerns, and ask and answer questions regarding their health. However, tracking more types of data did not necessarily transfer to useful *knowledge* that participants felt helped them to manage their health. Many participants mentioned that they were not sure about the relevance and usefulness of their data.

Although relatively rare, some researchers have examined the role of health literacy in the ability of individuals to track and monitor their health behaviors [47,48]. For example, Porter et al [47] conducted a diary study focused on people’s recording of their daily physical activities and found that highly health literate individuals were able to achieve a higher accuracy in their diaries. Zoellner et al [48] significantly reduced the sugar-sweetened beverage intake of the participants using a health literacy–centric intervention. Therefore, health literacy is seen as an essential *human tool* to support individuals in taking advantage of self-tracking technologies [49]. Although these studies did not examine how to improve the health literacy of individuals, we see opportunities to improve health literacy in people through their engagement in the very act of self-tracking itself and through feedback generated based on one’s personal health data. One opportunity is to deliver health knowledge relevant to individuals’ PGD. For example, food tracking apps, such as MyFitnessPal [50], suggest whether one’s food intake meets their nutritional needs when they record an entry and provide recommended dietary guidelines with feedback on one’s food consumption. Unlike traditional health literacy programs that focus on delivering general health knowledge [39,51], specific information related to an individual’s own health data can be easier to understand because it is tied to their own health concerns. However, we must keep in mind that interpretation of automatically tracked data is sometimes unreliable or may even cause misunderstandings [52,53]. For example, sleep tracking apps using pressure sensors to estimate sleep hours can cause false-positive detections when people are performing other activities in bed [53]. Therefore, in delivering health information based on an individual’s data to promote health literacy, it is important to ensure the accuracy of the data and to provide relevant and actionable advice.

### The Gaps Between Tracking and Sharing PGD

Our results show that patients who track their data do not always share those data with clinicians. Patients who shared their data during their clinic visits and those with a higher level of AE reported greater willingness to share their PGD with clinicians in the future. Although participants acknowledged several benefits of sharing their data with clinicians, over half of the participants reported one or more barriers to data sharing. First, participants were uncertain about the relevance of the data to their health or felt that their data were not relevant. Although not all types of PGD are necessarily related to patients’ immediate health concerns, these data can be important health indicators for preventative care [54-56]. For example, an individual’s activity level and caffeine consumption can affect their sleep quality [54]; similarly, changes in one’s heart rate may indicate a risk of cardiovascular disease [56]. To optimize the value of PGD, it is important to empower patients to share their data with clinicians when necessary. We believe that both people and technology play important roles in this process: clinicians can point out specific types of PGD that may be helpful for diagnosis and decision making; tracking tools can be designed to inform patients of how their PGD may or may not be relevant to their present health concerns. For example, by supporting self-experimentation, tracking tools can draw potential correlations between patients’ health indicators (eg, symptoms) and their daily behaviors [22]. These tools can also provide feedback based on the data and other contextual information of individuals to help them reflect on their behaviors and better assess the relevance of their data to their health [16].

Second, participants reported that sharing their PGD with clinicians was sometimes difficult because they did not have enough data. Some participants worried that their data were imprecise, whereas others lacked access to professional devices to measure important body metrics, such as heart rate and blood pressure. To encourage patients to share their PGD with clinicians, we argue that there is a need to better support people’s ability to capture sufficient and accurate PGD over time. Due to the high data capture burden [14] and lack of flexibility in existing tracking tools [13,14], many people cannot adhere to the tracking plans, and may even abandon tracking. To address these challenges, we call for flexible and low-burden tracking tools to better support the ability of patients to collect their health data over a long period, which can help both patients and clinicians. For example, semiautomated tracking tools can be used to lower data capturing burden by leveraging automatic tracking (eg, sleep data from Fitbit [57]) and enable flexible data capture through manual tracking (eg, mood) [58,59]. In addition, efforts need to be made to lower the barriers to tracking devices and to make them more accessible to the public (eg, setting up blood pressure kiosks).

Third, participants were reluctant to share their data because of the limited time they had to meet with their clinician. Participants explained that to make the best use of the time, they did not want to waste the time on sharing their PGD, especially when they considered that their data would not help clinicians. However, because patients are not medical experts, they could not always tell which data are relevant to their health, especially if they kept track of various types of data. Although our work did not examine clinicians’ perspectives on PGD sharing, previous work has found that clinicians are also concerned about the time they need to review PGD in the clinic [6,60] and may even consider these data to be distracting [52]. To resolve the dilemma of clinicians having limited time and patients feeling uncertain about whether their personal health data can be helpful, an approach should be developed to help patients share their data efficiently. For example, Fitbit recently rolled out a health and wellness report [61] that includes a visual or textual summary of a user’s data, which the individual can print out and take to the clinician’s office [4]. Although the relevance of these data to one’s health may be unclear to the patient, we believe that this is a promising step to support patients in sharing their data with their clinicians, leveraging consumer health technologies.

Finally, some participants reported that they were reluctant to share their PGD because of privacy concerns and fear of being judged, especially when asked to share data relating to unhealthy behaviors, such as smoking. This fear may stem from how tracking tools often pass judgment on people’s data, as researchers have noticed many self-tracking tools (eg, MyFitnessPal [50], Lose It! [62]) posit weight gain and extra calorie intake as negative outcomes (eg, using red to highlight these data points). Although such designs serve as an alert for individuals to maintain a healthy lifestyle, they can increase the anxiety of those who have weight concerns or dietary problems [63]. To help patients overcome their fear of being judged, tracking tools should avoid creating an environment that perpetuates stigma and that passes explicit or implicit judgments on users.

### Limitations and Future Work

This study has limitations that should be considered when interpreting the results and implications. First, although significant, the adjusted R-square statistics in our regression models were relatively low, with a medium effect size (Table 3), indicating that the models explained only a small proportion of the variances in our outcome variables (ie, HSI, AE, willingness to share one’s data with clinicians in the future). This could be partly because of either (1) omitting other confounding factors that could influence the health literacy of participants (eg, socioeconomic status, health insurance) [64,65] and data sharing practices (eg, the questions clinicians asked) or (2) sampling bias resulting from recruiting mostly young, healthy, and tech-savvy college students with adequate technology access and higher HSI and AE than patients in general [66,67]. Second, we characterized the tracking practices of participants based on the number of data types they tracked and how they tracked their data, which did not capture all dimensions related to self-tracking. Third, some participants reported sharing PGD they had not tracked (eg, blood pressure taken in the clinic), possibly because (1) they interpreted PGD as encompassing health-related data captured in the clinic, despite our best efforts to define and communicate its meaning or (2) they were able to retrospectively recall or estimate some forms of PGD, even if they had not explicitly tracked the data (eg, weight). Fourth, to make the survey easy and quick to respond to, we omitted questions inquiring about how the PGD sharing happened during the clinic visit. For example, we did not specifically ask participants what triggered their data sharing (eg, whether participants were asked by the clinician, had proactively shared their data themselves, or had their body metrics assessed in the clinic). In addition, we did not ask participants how they shared their data (eg, verbal communication, screen sharing). Finally, we conducted the survey at a UHC, where the clinicians might not be the primary physicians of the participants, which may limit the generalizability of our findings.

**Figure 1:**
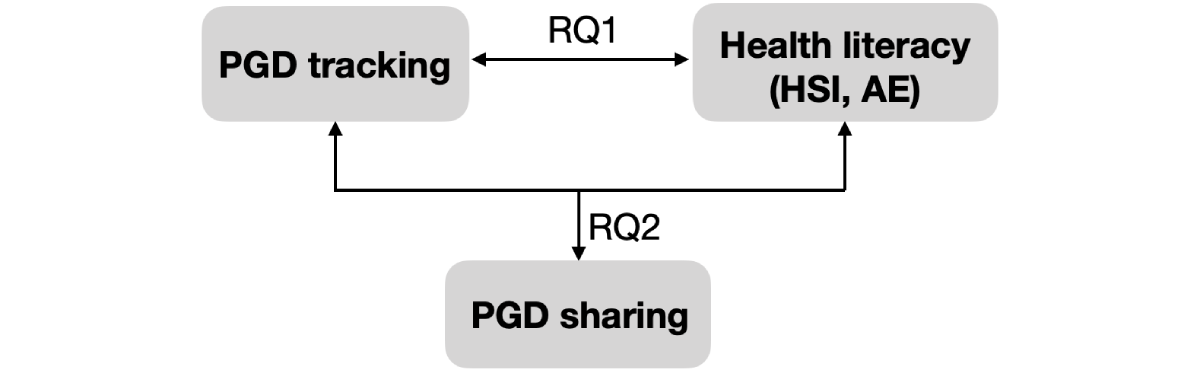
Interrelationships of PGD tracking, PGD sharing, and health literacy (HSI and AE). AE: the ability to actively engage with health care providers; HSI: having sufficient information to manage one’s health; PGD: patient-generated data; RQs: research questions.

Despite these limitations, we believe that, in optimizing health care for the next generation, investigating the interrelationships between PGD tracking and sharing practices, health literacy, and clinic experiences of tech-savvy college students is an important starting point. Going forward, we aim to further examine whether and how health literacy relates to different PGD tracking and sharing contexts—for example, replicating the study with different patient populations, such as those with more severe health conditions and those with low health literacy. Other dimensions of patients’ self-tracking practices, such as the frequency with which they track their data (eg, routine vs casual trackers), and triggers and methods of data sharing in the clinic warrant future research [68]. In addition, our study focused only on 2 health literacy constructs (HSI and AE). It is worth investigating how patients’ data tracking and sharing practices may relate to other health literacy constructs, such as one’s ability to navigate the health care system [11]. Finally, by surveying patients immediately after they met with a clinician, our study demonstrates the value of gathering immediate perspectives of patients on their experiences with PGD sharing, which allowed us to quantitatively explore the interrelationships between patients’ data tracking practices, data sharing practices, and their health literacy (Figure 1). In the future, similar approaches can be used to examine the immediate perspectives of clinicians on their experiences in responding to and using PGD shared by their patients.

### Conclusions

We presented an onsite survey study of 109 patients who had just met with a clinician at a UHC. Our aim was to investigate the potential relationships between patients’ PGD tracking practice, sharing practices, and their health literacy. We found that neither the number of data types the participants tracked nor how they tracked their data related to participants’ perceptions that they had sufficient information to manage their health (HSI). However, participants who reported tracking more types of PGD provided higher ratings when asked about their ability to actively engage with their health care providers. Our results also highlighted that tracking PGD does not always lead to sharing these data with clinicians, as exemplified by the many barriers participants reported regarding data sharing. Reflecting on our findings, we discussed ways to close the gaps between patient tracking and sharing of their PGD. We also suggested design opportunities to improve patient health literacy by leveraging the value of PGD to support patients in assessing the relevance of their PGD to their health and to facilitate patient data sharing with their clinicians.

## Appendix

Multimedia Appendix 1 Survey questionnaire.

## Abbreviations

AE: the ability to actively engage with health care providers
eHEALS: eHealth literacy scale
HLQ: health literacy questionnaire
HSI: having sufficient information to manage one’s health
IBS: irritable bowel syndrome
PGD: patient-generated data
RQ: research question
TOFHLA: Test of Functional Health Literacy in Adults
UHC: University Health Center
