# Supplementary material for: Interrelationships Between Patients’ Data Tracking Practices, Data Sharing Practices, and Health Literacy: Onsite Survey Study
Source: J Med Internet Res. 2020 Dec 22;22(12):e18937. doi: 10.2196/18937 (PMC7785405; doi:10.2196/18937)
Supplement: Multimedia Appendix 1 [file jmir_v22i12e18937_app1.docx]

## Multimedia Appendix: Survey Questions

What is Personal Health Data? In this survey, we use the term “Personal Health Data” to refer to health-related data that people keep track of about themselves, such as exercise (e.g., step count, miles run), sleep, diet, heart rate, and blood pressure data.

In this survey, you will be asked questions about your basic demographics, your personal health data tracking, and your experience of communicating personal health data with your doctors. Please answer the following questions.

Pleases answer the following questions.

1. Your Age: ( )
2. Gender:
   - Male
   - Female
   - Non-binary/third gender
   - Prefer to self-describe: _____________________
   - Prefer not to say
3. What is your race?
   - Asian
   - Black or African-American
   - White
   - Native Hawaiian or other Pacific Islander Multiracial
   - Other, please specify: _____________________
4. What is your ethnicity?
   - Hispanic or Latino
   - Not Hispanic or Latino
   - Prefer not to say
5. What is your first language? ( )
6. In which country were you born? ( )
7. How long have you been in U.S.?
   - Less than 1 month
   - 1 month to less than 3 months
   - 3 months to less than 6 months
   - 6 months to less than 1 year
   - 1 year to less than 2 years
   - 2 year to less than 3 years
   - 3 years to less than 4 years
   - 4 years to less than 5 years
   - 5 years to less than 6 years
   - 6 years to less than 7 years
   - 7 years to less than 8 years
   - 8 years to less than 9 years
   - 9 years to less than 10 years
   - 10 years or more
8. What is your highest level of educational attainment?
   - Some high school or less
   - High school diploma
   - General Educational Development (GED) Some college
   - Associate’s (2-year) degree
   - Bachelor’s degree
   - Some graduate or professional school
   - Graduate or professional degree
   - Other (please specify): __________________
   - Prefer not to say
9. What is your current employment status?
   - Employed
   - Unemployed
   - Disabled
   - Retired
   - Student
   - Homemaker
   - Prefer not to say
10. What is your approximate household income?
    - Less than $30,000/year
    - $30,000–$49,999
    - $50,000–$74,999
    - $75,000+
    - Prefer not to say
11. Would you say that in general your health is:
    - Poor
    - Fair
    - Good
    - Very Good
    - Excellent
12. In general, how much control do you feel you have over your health?
    - None at all
    - Very little
    - Neutral
    - Some
    - A great deal
13. Do you personally own any of the following (Check all that apply):
    - Desktop computer
    - Laptop computer
    - Tablet
    - Smartphone
    - Personal health tracking device (e.g., Fitbit, Apple Watch)
    - Medical device (e.g., Glucometer, home blood pressure monitor)
14. Do you have Internet access at home?
    - Yes (Broadband/Cable)
    - Yes (Dial-up)
    - No
15. How frequently do you use the Internet to look for health-related information?
    - Never
    - Rarely
    - Sometimes
    - Frequently
    - Very frequently

Please answer how strongly you disagree or agree with the following statements:

1. I feel I have good information about health.
   - Strongly disagree
   - Disagree
   - Agree
   - Strongly agree
2. I have enough information to help me deal with my health problems.
   - Strongly disagree
   - Disagree
   - Agree
   - Strongly agree
3. I am sure I have all the information I need to manage my health effectively.
   - Strongly disagree
   - Disagree
   - Agree
   - Strongly agree
4. I have all the information I need to look after my health.
   - Strongly disagree
   - Disagree
   - Agree
   - Strongly agree

Please answer how easy or difficult the following tasks are for you to do:

1. Make sure that healthcare providers understand your problems properly.
   - Cannot do
   - Very difficult
   - Quite difficult
   - Quite easy
   - Very easy
2. Feel able to discuss your health concerns with a healthcare provider.
   - Cannot do
   - Very difficult
   - Quite difficult
   - Quite easy
   - Very easy
3. Have good discussions about your health with doctors.
   - Cannot do
   - Very difficult
   - Quite difficult
   - Quite easy
   - Very easy
4. Discuss things with healthcare providers until you understand all you need to.
   - Cannot do
   - Very difficult
   - Quite difficult
   - Quite easy
   - Very easy
5. Ask healthcare providers questions to get the health information you need.
   - Cannot do
   - Very difficult
   - Quite difficult
   - Quite easy
   - Very easy

Please answer the following questions.

1. What kinds of personal health data do you currently keep track, if any? (Check all that apply)
   - Exercise (e.g., step count, miles walked/run)
   - Sleep
   - Weight
   - Food intake,
   - Water intake
   - Sedentary (sitting) time
   - Mood
   - Stress
   - Heart rate
   - Blood pressure
   - Cholesterol
   - Blood glucose
   - Other (Please specify): ___________________
   - None (I currently do not keep track of any personal health data.)
2. How do you keep track of your personal health data, if any?
   - Paper, like a notebook or journal
   - A computer program, like a spreadsheet
   - A website or other online tool
   - An app on your phone or mobile device
   - Wearables, like a wristband or clip-on
   - A medical device, like a glucometer or blood glucose meter
   - In my head
   - Other (Please specify): __________________
   - Not applicable (I don’t keep track of my personal health data.)
3. What is the reason for your visit today?
   - Regular check up
   - Inquiry/exam for a specific injury, illness, or condition
   - Follow-up visit
   - Other (Please specify): __________________
4. How long was your face-to-face meeting time with your doctor today?
   - Less than 5 minutes
   - 5 to less than 10 minutes
   - 10 to less than 15 minutes
   - 15 to less than 20 minutes
   - 20 to less than 25 minutes
   - 25 to less than 30 minutes
   - 30 minutes or longer
5. Do you feel that this amount of time was enough to meet your needs?
   - Yes
   - No
   - Unsure
6. Overall, how satisfied or dissatisfied were you with your meeting with your doctor today?
   - Very Dissatisfied
   - Somewhat Dissatisfied
   - Neutral
   - Somewhat Satisfied
   - Very Satisfied
7. Could you tell us what questions your doctor asked you today (if you are willing to share)?
   _____________________________________________________________
8. How easy or difficult was it to answer your doctor's questions (if any)?
   - Very Difficult
   - Somewhat Difficult
   - Neutral
   - Somewhat
   - Easy
   - Very Easy
9. What kinds of personal health data did you share (e.g., showing data/print out, or just verbally) with your doctor today, if any? (Check all that apply)
   - None (I didn’t share any of my personal health data with my doctor today.)
   - Exercise (e.g., step count, miles walked/run)
   - Sleep
   - Weight
   - Food intake
   - Water intake Sedentary (sitting) time Mood
   - Stress
   - Heart rate
   - Blood pressure Cholesterol
   - Blood glucose
   - Other (Please specify): __________________
10. What barriers (if any) do you have when sharing your personal health data with your doctor? (Check all that apply)
    - I do not share my personal health data with my doctor.
    - I do share my personal health data with my doctor, but I have not encountered any barriers at all.
    - I am not sure how to talk to my doctor about my personal health data
    - I am not sure if sharing my personal health data would help my doctor.
    - There is not a good way to share my personal health data, although I would like to.
    - I do not have enough data
    - My personal health data is irrelevant to my current illness or condition.
    - I am not sure what kinds of personal health data might be important to share regarding my illness or condition.
    - My time with the doctor is limited during the appointment.
    - I am not sure if my doctor would accept/welcome my data sharing.
    - I am concerned about my privacy
    - I am worried about what my doctor might do with my data.
    - Other (Please specify): ___________________
11. Could you tell me a little more about your answer choice(s) above?

_____________________________________________________________

1. List up to 3 personal health data types (such as step counts, food intake, or blood sugar readings) that you think it would have been helpful if you had tracked them and shared them with your doctor today.

_____________________________________________________________

1. Please explain how you think sharing of the personal health data (mentioned above) would have been helpful during your visit today.

_____________________________________________________________

1. How likely are you to share your personal health data in future doctor visits?
   - Very unlikely
   - Somewhat unlikely
   - Neutral
   - Somewhat likely
   - Very likely
2. Please explain your response to the question above.

_____________________________________________________________
